# Mitigating Benzodiazepine Dependence and the Risk of Drug-Induced QTc Prolongation in the Treatment of Gastroparesis: A Case Report

**DOI:** 10.3390/medicina58030409

**Published:** 2022-03-10

**Authors:** Karley Tranchina, Derek Matlock, Carlos Hernandez, Jacques Turgeon, Jennifer M. Bingham

**Affiliations:** 1 College of Pharmacy, University of Arizona, Tucson, AZ 85721, USA; ktranchina@pharmacy.arizona.edu; 2 MedWiseRx, 100 N Stone Ave., Tucson, AZ 85701, USA; damatlock@trhc.com (D.M.); cjhernandez@trhc.com (C.H.); 3 Tabula Rasa HealthCare Group, Precision Pharmacotherapy Research and Development Institute, 13485 Veterans Way, Orlando, FL 32827, USA; 4 Tabula Rasa HealthCare Group, Office of Translational Research and Residency Programs, 228 Strawbridge Dr, Moorestown, NJ 08057, USA; jbingham.pharmd@gmail.com

**Keywords:** benzodiazepine dependence, QTc prolongation, pharmacist, medication safety, gastroparesis

## Abstract

Patients are often faced with challenges when it comes to safe therapeutic options. An 89-year-old female with a history of arrhythmias and refractory gastroparesis complained of adverse drug events from her benzodiazepine. While performing a comprehensive medication review and a medication safety review using an advanced clinical decision support system, the pharmacist successfully tapered off the benzodiazepine to a safer alternative antidepressant indicated for the treatment of gastroparesis. Special attention was given to selecting drugs with less QT prolongation risk, based on her age, current drug regimen, previous medical history, and presence of polypharmacy.

## 1. Introduction

Gastroparesis is an uncommon disease that results from abnormal or absent stomach motility, resulting in incomplete digestion [1]. While there are numerous medications that delay gastric emptying and contribute to gastroparesis, there are limited therapeutic options that do not increase the risk of QTc prolongation or fatal arrythmias. The following case presentation describes a geriatric patient treated for refractory idiopathic gastroparesis with a long history of benzodiazepine use (leading to benzodiazepine dependence) and at potential risk of QT prolongation associated with her drug regimen. 

## 2. Case Presentation

An 89-year-old female with a past medical history of gastroparesis with nausea, asthma, hip fracture, right hip replacement, and degenerative/scoliosis of the lumbar spine was consulted by the pharmacist. During the initial consultation, the pharmacist observed that the patient had not achieved adequate symptom relief with her current gastroparesis regimen (Table 1). 

A past medication history revealed that the patient was first trialed, over 20 years ago, on domperidone for idiopathic gastroparesis. She had no history of infection or post-infectious state. Her HASBLED score revealed a low risk for major bleeding (i.e., one point). 

Prior to the pharmacists’ consultation, the patient had solely received care for the past 20 years from a homeopathic provider and went without cardiac monitoring. Upon reviewing her patient medical history and according to the patient, domperidone was discontinued due to unspecified cardiac arrhythmias. Next, she was prescribed erythromycin, which was discontinued due to uncontrollable diarrhea. Then, she was prescribed lorazepam. Over time, her tolerance to lorazepam increased, leading to larger doses and dependence. 

During the initial pharmacist consultation, she expressed concern about her dependence on benzodiazepines, and she indicated that she was afraid to experience symptoms of withdrawal if she missed one dose. The patient was taking lorazepam (1 milligram by mouth three times a day) to symptomatically treat the nausea and anxiety associated with gastroparesis. As the pharmacist was addressing the benzodiazepine dependence and looking for alternatives, she performed a comprehensive medication review and a medication safety review of the drug regimen using an advanced clinical decision support system (CDSS). The objective was to make a holistic assessment of her condition, drug history, and drug regimen appropriateness, intervene in and address her benzodiazepine dependence, and reduce her risk of falls, QT prolongation, and fatal arrythmias. The CDSS (i.e., MedWise) used proprietary algorithms to analyze medication-related factors and helped the pharmacists to determine the risk of such adverse drug events. [2] The patient consented to deprescribing her lorazepam and slowly tapering off the dose to avoid hospitalizations, emergency department visits, and other medication-related problems. Furthermore, her risk of QT prolongation was increased by the unmodifiable risk factors of advanced age and female sex, on top of suspected events and combined drug therapy.

The patient’s benzodiazepine was tapered slowly according to parameters set forth by the EMPOWER Trial [3]. The pharmacist initiated a 25% decrease every 2 weeks. Given the patient’s age and to avoid withdrawal symptoms, the pharmacist was cognizant of the need to lengthen the taper to every 3 weeks. As the dose of lorazepam was slowly decreased, the pharmacist continued to re-assess the patient for gastroparesis symptom management at each follow up visit. In addition, the pharmacist continuously researched safer alternative therapeutic options for gastroparesis in geriatric patients. Due to the risk of QTc prolongation with many of the prokinetic agents, a limited number of options existed for this patient. 

After 22 weeks, the patient was successfully tapered off lorazepam (Table 2). She was then started on mirtazapine 7.5 milligrams taken by mouth every night at bedtime. Next, this was increased to 15 milligrams by mouth at bedtime after two weeks. The patient tolerated this dose well without any adverse drug effects. The use of mirtazapine decreased patient anxiety surrounding her condition, as well as her insomnia. Lastly, it provided some benefit for her reported nausea, which was the most concerning gastroparesis symptom per the patient. It is important to note that there have also been cases of torsade de pointes (TdP) reported with mirtazapine, typically in overdose situations, and use of the drug is considered to place patients at moderate risk [4]. Therefore, the pharmacist recommended that the patient continued to be monitored for QTc prolongation through use of electrocardiograms and electrolyte evaluation every 3–4 months [5].

When developing this plan, the pharmacist utilized evidence from other case reports and studies to determine that mirtazapine might be an appropriate alternative. Marella et al. found that 15 mg of mirtazapine administered nightly resulted in symptom relief for a patient with diabetic gastroparesis [6]. An open-label trial looked at the use of 15 mg mirtazapine nightly in 30 individuals with refractory gastroparesis, and found that it significantly improved nausea and vomiting at 2 and 4 weeks [7]. The patient’s risk of QT prolongation, multiple failed attempts with other prokinetic agents, and concomitant anxiety is what led the pharmacist to ultimately make the decision to trial mirtazapine. 

## 3. Drug-Induced QT Prolongation Concerns

Long QT syndrome is a cardiac condition where the repolarization phase of ventricular cardiomyocytes is prolonged [8]. This condition is reflected in the surface electrocardiogram (ECG) by a prolongation of the QTc interval. The condition may be asymptomatic for a long time, but under extreme conditions early afterdepolarizations may develop, potentially leading to a polymorphic ventricular tachycardia, known as TdP, and sudden death [8,9]. Long QT syndrome can be inherited (i.e., congenital disease) or caused by medications, electrolyte imbalances, or other medical conditions [10,11]. The following medications are commonly used to treat gastroparesis, but evidence demonstrates that their use may lead to long QT syndrome. 

### 3.1. Domperidone

This medication is a peripheral dopamine D2-receptor antagonist that was previously indicated as a prokinetic and antiemetic agent [12]. However, studies have shown that domperidone may prolong cardiac repolarization by blocking the rapid component of the delayed rectifier potassium current (IKr), a major repolarization current in cardiac ventricular myocytes [12]. Drolet et al. found that 100 nanomoles/liter of domperidone prolonged cardiac repolarization by about 25–30% [12]. In a systematic review and meta-analysis, researchers determined that domperidone increased the risk of arrhythmia and sudden cardiac death by 70% [13]. Additional studies indicated the limited benefits of domperidone, considering the risk of toxicity and sudden death, and reported that use should be discouraged in geriatric patients [13,14,15]. This drug was not a viable option, as the patient reported treatment failure and adverse drug events with previous use. 

### 3.2. Metoclopramide 

Metoclopramide also blocks dopamine D2-receptors, as well as serotonin receptors in the central nervous system (CNS) at higher doses [16]. It is used to treat gastroparesis given its ability to enhance the response to acetylcholine in the gastrointestinal (GI) tract, thus, enhancing gut motility and gastric emptying [16]. Ellidokuz et al. completed a small double-blind placebo-controlled study in healthy male patients that evaluated the effects of metoclopramide on the QTc interval. They concluded that metoclopramide increased the QT/RR slope and QTc variance [17]. When the QT/RR slope is steep, this indicates changes in the QTc interval, including QTc prolongation [18]. CredibleMeds categorizes this medication as a drug with ‘Conditional Risk’, while the long QT-JT index lists metoclopramide as a high-risk medication [11]. Although the patient did not trial this medication, the pharmacist concluded that it would be less than favorable, considering the patient’s age and previous medical history. 

### 3.3. Erythromycin

This antibiotic is shown to improve gastric emptying in patients with gastroparesis [19]. However, it has also been associated with QTc prolongation, TdP, and other arrhythmias [20]. Studies have demonstrated that the QTc-prolonging effects of erythromycin are likely due to its inhibition of cardiac potassium currents [21]. Erythromycin blocks the potassium current of the cardiac cells, causing this adverse drug event [21]. Although the patient did try erythromycin in the past, it was discontinued due to complaints of diarrhea and was not considered favorable to trial again.

### 3.4. Cisapride

Cisapride is another GI prokinetic agent [22]. Its mechanism of action involves the release of acetylcholine at the myenteric plexus, the major nerve supply to the GI tract [22]. It has also been shown to be one of the most potent non-antiarrhythmic IKr blockers; it has a potency similar to terfenadine (removed from the market because of TdP) and is 10-times more potent than domperidone [23,24]. This results in a lengthening of cardiac repolarization as well as QTc prolongation [23]. In many countries, cisapride use is banned due to the risk of TdP and sudden death [25]. 

### 3.5. Mirtazapine

Lastly, mirtazapine is an antidepressant that antagonizes 5-HT2, 5-HT3, alpha2-autoreceptors and alpha 2-heteroreceptors to release norepinephrine, as well as histaminic H1 receptors, which lead to increased appetite and sedative effects. The blockade of 5-HT3 receptors results in decreased nausea [5]. However, mirtazapine has also been found to prolong the QTc interval and potentially result in TdP [4]. According to the package insert, studies have shown that the mean change in the QTc interval for patients taking mirtazapine was +1.6 milliseconds, while placebo was −3.1 milliseconds [26]. The Long QT-JT index classifies mirtazapine as a drug with moderate risk [11]. For additional risk factors for long QT syndrome, see Table 3.

## 4. Conclusions

When it comes to the treatment of gastroparesis, few agents can be used safely in geriatric patients. Further, when patient age, sex, and medical history are considered, this list becomes even shorter. For our patient, the pharmacist tapered the benzodiazepine and trialed a low dose of mirtazapine to circumvent the risk of QTc prolongation and falls, while treating gastroparesis and other comorbidities. In conclusion, the patient was successfully tapered off lorazepam after 22 weeks and low-dose mirtazapine aided in reducing the patient’s symptoms of anxiety related to gastroparesis, decreasing nausea, and better-quality sleep.

## Figures and Tables

**Table 1:** Medication List at Initial Consult.

Drug	Dose	Route	Frequency	Indication
Advair HFA	112 mcg/act	Inhalation	Twice daily	Asthma
Amylase	120,000	Oral	Three times daily	Digestion
Armour Thyroid	60 mg	Oral	Daily	Hypothyroidism
Cholecalciferol	2000 international units (50 mcg)	Oral	Daily	Osteoporosis
CoQ10	400 mg	Oral	Daily	Supplement
Gabapentin	200 mg	Oral	Daily at bedtime	Pain
Lorazepam	1 mg	Oral	Three times daily	Gastroparesis
Melatonin	10 mg	Oral	Daily at bedtime	Insomnia

Key: mcg = microgram; act = actuation; mg = milligram.

**Table 2:** Lorazepam Taper.

Week	Dose 1	Dose 2	Dose 3
0	1 mg	1 mg	1 mg
1 and 2	0.5 mg	1 mg	1 mg
3 and 4	0.5 mg	0.5 mg	1 mg
5 and 6	0.5 mg	0.5 mg	0.5 mg
7 and 8	0.5 mg	0.5 mg	-
9 and 10	0.25 mg	0.75 mg	-
11 and 12	0.25 mg	0.5 mg	-
13 and 14	0.25 mg	0.25 mg	-
15 and 16	0.5 mg/0.25 mg alternating days	-	-
17 and 18	0.25 mg	-	-
19 and 20	0.25 mg Drug free Mon/Wed	-	-
21 and 22	0.25 mg Drug free Mon/Wed/Fri	-	-

Key: mg = milligram; Mon = Monday; Wed = Wednesday; Fri = Friday.

**Table 3:** Risk Factors for Long QT Syndrome [27].

Female Sex	Advanced Age	Bradycardia	Electrolyte Imbalance	Concomitant Diseases
Women at a 2- to 3-fold risk	↓ drug metabolism Polypharmacy ↑ drug-drug interactions	SA node polymorphisms contribute to bradycardia Medications (e.g., diltiazem, digoxin, beta blockers, clonidine)	↓ potassium ↓ magnesium ↓ calcium ↓ available open channels and ↑ channels blocked	Myocardial infarction, heart failure, left ventricular hypertrophy, eating disorders, kidney disease, liver disease

## Data Availability

The data presented in this study are available in the article.
